# Factors associated with HIV self-testing among female university students in Uganda: a cross-sectional study

**DOI:** 10.1186/s12981-022-00484-x

**Published:** 2022-12-01

**Authors:** Ivan Segawa, Sabrina Bakeera-Kitaka, Kenneth Ssebambulidde, Timothy R. Muwonge, Lorraine Oriokot, Kevin Ouma Ojiambo, Andrew Mujugira

**Affiliations:** 1 Clinical Epidemiology Unit, College of Health Sciences, Makerere University, P.O.Box 7072, Kampala, Uganda; 2 Department of Paediatrics, College of Health Sciences, Makerere University, Kampala, Uganda; 3 Infectious Diseases Institute, College of Health Sciences, Makerere University, Kampala, Uganda; 4 The Campbell Collaboration, Oslo, USA

**Keywords:** Women, Universities, HIV, Self-testing, HIV testing, Students

## Abstract

**Background:**

Adolescent girls and young women (AGYW) at institutions of higher learning are at high risk of HIV, and conventional HIV testing services may not reach them sufficiently. HIV self-testing (HIVST) scalability can be informed by identifying AGYW who have used or are interested in using HIVST. We aimed to determine factors associated with use and willingness to use HIVST among female university students.

**Methods:**

An online cross-sectional survey was conducted among 483 female students at Makerere University, Uganda. Proportions of students who have used or are willing to use HIVST and their associated factors were determined. Modified Poisson regression models were used to estimate prevalence ratios (PR) and their 95% confidence intervals (CI).

**Results:**

The median age of the participants was 22 (Interquartile range [IQR] 21–23) years, and 21% had never tested for HIV. Over 93% were willing to utilize HIVST, and 19% had ever used HIV self-test kits. Increasing age (adjusted prevalence ratio [aPR] 1.23 per year, 95% CI 1.07–1.43) was significantly associated with HIVST use. Predictors of willingness to self-test for HIV were college type (arts vs. science-based, aPR 0.92, 95% CI 0.88–0.97), number of sexual partners (one, aPR 1.07, 95% CI 1.03–1.12 or ≥ 2, aPR 1.08, 95% CI 1.04–1.19, vs. none), alcohol (aPR 1.04, 95% CI: 1.00–1.09) or injection drug (aPR 1.04, 95% CI 1.00–1.09) use, a history of sexually transmitted infections in past 12 months (aPR 1.05, 95% CI 1.01–1.09), and HIV testing experience (tested in past 12 months, aPR 1.12, 95% CI 1.02–1.22 or over 12 months, aPR 1.13, 95% CI 1.03–1.24, vs. never tested).

**Conclusion:**

HIVST was highly acceptable despite its limited use. This study demonstrates female student characteristics that can be leveraged to scale up HIVST programs in higher institutions of learning.

## Introduction

Over 4900 adolescent girls and young women (AGYW), aged 15–24 years, acquired HIV every week in 2021, with 82% residing in sub-Saharan Africa [1]. With 212 AGYW acquiring HIV every week [2], Uganda is one of the top 20 countries with the highest HIV incidence rate among this population [3]. HIV prevalence among older AGYW 20–24 years (4.2%) is more than double that among AGYW 15–19 years (1.7%) [4]. Thus, female university students, typically aged 20–24 years in Uganda, are at high risk of HIV infection [5, 6]. Despite the high HIV burden among AGYW in Uganda, the HIV prevalence of female university students (1.5%) [7] is nearly 50% lower than the national AGYW prevalence of 2.9% [4].

According to the 2016–2017 Uganda Population-based HIV Impact Assessment, more than half (54–60%) of AGYW living with HIV were unaware of their HIV status [8]. Specifically, only 74% of female students in six Ugandan universities had tested for HIV [6]. In this setting, HIV risk increased with higher education attainment and was higher among urban residents [9]. Although current data on HIV test uptake by young women attending universities in Uganda is scarce, it is likely to fall short of the Joint United Nations Programme on HIV/AIDS (UNAIDS) goal of 95% of all people with HIV knowing their HIV status by 2025 [10]. HIV testing is the entry point to care and prevention services [11], but conventional HIV testing services have not been effective at reaching undiagnosed high-risk populations [11].

In 2016, the World Health Organization (WHO) recommended HIV self-testing (HIVST) as an additional testing strategy to reduce HIV testing gaps, especially among high-risk individuals [12]. HIVST increases testing uptake and is not associated with increased sexual risk behaviour among key populations [12]. The Swipe And Know Your Status (SAYS) HIVST initiative, for example, increased HIV testing services among university students in Zimbabwe from 6% in the previous year to 44% in just 6 months [13]. AGYW have exhibited high acceptability and demand for HIVST due to convenience, non-invasive approaches, and privacy, and ability to self-test and interpret their results accurately [13–15]. HIVST users often prefer HIVST to blood-based HIV testing because it is painless and easy to use [15]. Some AGYW may however be reluctant to use HIV self-test kits because of high cost of the kits, perceived inaccuracy of the results, and missed opportunities for face-to-face counselling and linkage to care [15, 16].

AGYW with a history of use of HIVST can be trained and leveraged as peers to mobilize colleagues to self-test, distribute kits, and support those eager to self-test for HIV [13, 17]. Formative surveys can identify peers before implementing HIVST scale-up programs [13]. Identifying characteristics of AGYW willing to use HIVST also helps prioritize distribution of self-test kits among peers during HIVST expansion and scale-up programs. We aimed to assess factors associated with use and willingness to use HIVST among female university students at a public university in Uganda.

## Methods

### Study design and setting

From 1st to 26th October 2021, we conducted an online cross-sectional survey among female students at Makerere University in Kampala, Uganda. Makerere University has 43,000 undergraduate and 6000 postgraduate students (overall 44% are female) representing about one third of enrolled university students in Uganda. The survey was conducted during the COVID-19 lockdown period in Uganda, when most student learning and access was virtual. Free HIV counselling and testing services, including HIVST for students and staff, are available at Makerere University Hospital.

### Participants and procedures

Female undergraduate students aged 15–24 years, with a self-reported HIV negative status, and who consented to participate met the study eligibility criteria. Students also had to be enrolled at Makerere University in Kampala and reachable through social media or email during the study period. Female students were selected using the quota sampling technique. The quotas were the 10 colleges at Makerere University, and students were consecutively selected from the different schools, courses, and years of study in each college. Research assistants obtained telephone numbers of female students from class representatives. Eligible students were contacted and asked if they were willing to participate in the study.

The study team received protocol training on use of data collection tools prior to study onset. The questionnaire was pilot tested among 21 female students to inform and modify the data collection form and processes, especially skip logic patterns. These students were excluded from the study using email address verification. The study team contacted eligible study participants and provided a link to the online survey (Google Forms, Google Inc., Mountain View, CA, USA) through email or WhatsApp (WhatsApp Inc., Menlo Park, CA, USA), according to their preference. Prior to answering the questionnaire, study participants were asked to provide informed consent. The consent form included a summary of the study including potential risks and benefits and was attached to the online survey. Clicking a button at the end of the consent form indicated consent to participate in the study. Participants were reimbursed with UGX 20,000 (~ $5) in accordance with local ethics committee guidance. Young women found to be at high risk for HIV were referred to the University Hospital for risk counselling and access to HIV prevention methods.

### Statistical analysis

The primary outcomes were a history of HIV self-test use (yes/no) and willingness to use HIVST (yes/no). The question “Are you willing to use HIV self-test kits when freely available at your university, hostel, or residence?” was used to assess willingness to use HIVST. HIVST use was reported as a proportion with 95% confidence intervals (CI) after adjusting for clustering (college as strata). The study had 80% power to detect a significant difference in history of use of HIVST among women that had never tested (π_1_ = 0.181) and those that had at least one HIV test in the past 12 months (π_2_ = 0.449) [18, 19] with an initial sample size of 192 participants. A final sample size of 427 participants was considered for this sub-study, assuming a design effect of 2, a 90% response rate, and a two-sided alpha of 0.05. Considering that this was a subset of a larger study, the largest sample size of 516 participants was utilized for the overall study. However, because the design effect of the study was found to be approximately one, we did not adjust for clustering in further analysis. Numerical variables were summarized using means (and standard deviations) or medians (and interquartile ranges), and categorical variables using proportions. We used a modified Poisson regression model with robust standard errors to assess factors independently associated with use and willingness to use HIVST. The regression models reported prevalence ratios (PR) and their 95% CI. Covariates included socio-demographic, academic, and economic characteristics, and sexual risk behaviours; factors with p < 0.20 were included in multivariable models. Interaction among variables was assessed using the chunk test. Variables which changed the PR by ≥ 10% were considered potential confounders. Using a manual stepwise selection, we retained variables in the final model with p-values < 0.05 and their potential confounders. Responses with missing outcomes were excluded in analysis. Data were analysed using STATA 14 (StataCorp, College Station, TX, USA).

### Ethical considerations

Ethical approval was obtained from Makerere University School of Medicine Research Ethics Committee (Mak-SOMREC-2021–181). Administrative clearance to conduct the study was obtained from the Clinical Epidemiology Unit of Makerere University.

## Results

### Demographic characteristics

A total of 534 young women participated in the study. We excluded 51 from the analysis: 24 were older than 24 years, 12 were male, 8 did not consent, and 7 were duplicate entries. For the remaining 483, the median age was 22 years (interquartile range [IQR] 21–23; Table 1). Of these, 53% (254) had ever had sex, 29% (139) had used emergency contraception in the prior 6 months, 21% (100) had never tested for HIV, 21% (97) had partners > 10 years older, 12% (57) had multiple partners, and 7% (34) had ever been pregnant. Ninety three percent (444/479) of students were willing to use HIVST if it was made available at no cost at their halls of residence or hostels, but only 19% (93) had ever used HIVST.

**Table 1:** Demographic Characteristics of Female Students (N = 483)

Characteristic	N (%)
Age (years), median (IQR)	22.2 (21.3–23.1)
Ever had sex	
No	229 (47.4)
Yes	254 (52.6)
Current sexual partners	
None/sexually inactive	234 (48.5)
1	227 (47.0)
2	19 (3.9)
> 2	3 (0.6)
Sexual relationship with more than 1 person in the past 6 months	
No	426 (88.2)
Yes	57 (11.8)
Partner is circumcised (N = 480)	
No	34 (7.1)
Yes	199 (41.5)
I don’t know	13 (2.7)
No sexual partner	234 (48.8)
Partner’s HIV status (N = 479)	
Negative	211 (44.1)
Positive	3 (0.6)
I don’t know	31 (6.5)
No sexual partner	234 (49.9)
Age of partner (N = 463)	
< 10 years from my current age	132 (28.5)
≥ 10 years from my current age	97 (21.0)
No sexual partner	234 (50.5)
Ever been or currently pregnant	
No	438 (90.7)
I don't know	11 (2.3)
Yes, currently pregnant	4 (0.8)
Yes, I’ve been pregnant before	30 (6.2)
Desired to become pregnant in the past 12 month	
No	434 (89.9)
Yes	49 (10.1)
Contraception used (multiple responses)	
None	233 (48.2)
Injectable	9 (1.9)
Implant	6 (1.2)
Oral contraceptive pills	53 (11.0)
IUD	4 (0.8)
Male condoms	91 (18.8)
Female condoms	8 (1.7)
Natural	46 (9.5)
Abstinence	139 (28.8)
Emergency contraceptive use in the past 6 months	
No	344 (71.2)
Yes	139 (28.8)
Ever been sexually assaulted (rape and defilement)	
No	439 (90.9)
Yes	44 (9.1)
Ever tested for HIV	
Never	100 (20.9)
Yes, within the past 12 months	215 (44.9)
Yes, more than 12 months ago	164 (34.2)
Self-perceived HIV risk	
None	229 (47.4)
Low	207 (42.9)
High	47 (9.7)

### Factors associated with use of HIV self-testing

In bivariate analysis, we found that increasing age, studying in an arts-based college, history of pregnancy, emergency contraceptive use, having one sexual partner, sexual experience, alcohol use, a history of unprotected sex with a partner of unknown HIV status, transactional sex, HIV post-exposure prophylaxis use, and fertility intentions were significantly associated with a history of use of HIVST (p < 0.05 for all comparisons; Table 2). Increasing age was significantly associated (adjusted prevalence ratio [aPR] 1.23 per year, 95% CI 1.07–1.43, p = 0.01) with HIVST use in multivariable analysis. Emergency pill use confounded the relationship between sexual experience (ever had sex) and HIVST use.

**Table 2:** Factors associated with HIVST use

Characteristic	Used (N = 93), N (%)	Crude PR^a^ (95% CI)	p value	Adjusted PR^a^ (95% CI)	p value
Age, years: Mean(SD)	22.6 (1.2)	1.30 (1.12–1.50)	** < 0.01**	1.23 (1.07–1.43)	**0.01**
Study year					
Year 1 & 2	49 (17.0)	Reference			
Year 3, 4 & 5	44 (23.2)	1.37 (0.95–1.97)	0.09	**–**	**–**
College type					
Science	21 (14.0)	Reference			
Arts	72 (21.9)	1.56 (1.00–2.44)	**0.05**^**b**^	**–**	**–**
Pregnancy history					
No	82 (18.4)	Reference			
Yes	11 (32.4)	1.76 (1.04–2.97)	**0.04**	**–**	**–**
Emergency pill in 6 months					
No	55 (16.1)	Reference		Reference	
Yes	38 (27.5)	1.71 (1.19–2.46)	** < 0.01**	1.33 (0.89–1.98)	0.16
Number of sexual partners					
None	32 (13.8)	Reference			
One	55 (24.4)	1.77 (1.19–2.63)	**0.01**	**–**	**–**
Two or more	6 (27.3)	1.98 (0.93–4.21)	0.08	**–**	**–**
Ever had sex					
No	30 (13.2)	Reference		Reference	
Yes	63 (25.1)	1.91 (1.28–2.83)	** < 0.01**	1.54 (0.99–2.40)	0.05
Alcohol use in past 3 months					
No	62 (17.4)	Reference			
Yes	31 (25.4)	1.46 (1.00–2.14)	**0.05**^**b**^	**–**	**–**
Unprotected sex with > 1 partner of unknown HIV status					
No	84 (18.6)	Reference			
Yes	9 (33.3)	1.79 (1.02–3.16)	**0.04**	–	–
Anal sex in the past 6 months					
No	85 (18.9)	Reference			
Yes	8 (28.6)	1.51 (0.82–2.81)	0.19	–	–
Transactional sex in past 6 months					
No	83 (18.5)	Reference			
Yes	10 (33.3)	1.80 (1.04–3.10)	**0.03**	–	–
Injection drug use in the past 6 months					
No	90 (19.1)	Reference			
Yes	3 (37.5)	1.96 (0.79–4.90)	0.15	–	–
STI in the past 12 months					
No	65 (17.8)	Reference			
Yes	28 (24.8)	1.40 (0.94–2.06)	0.09	–	–
PEP^c^ use in the past 12 months					
No	86 (18.7)	Reference			
Yes	7 (38.9)	2.08 (1.13–3.84)	**0.02**	–	–
Desired to become pregnant in the past 12 months					
No	77 (17.9)	Reference			
Yes	16 (33.3)	1.87 (1.19–2.92)	**0.01**	–	–

^a^PR (Prevalence Ratio);

^b^p = 0.049;

^c^HIV post-exposure prophylaxis (PEP);

Partner has other partners, partner is circumcised, partner provides financial and material support, partner’s HIV status, partner among populations at high risk, and age of partner (all p < 0.05 for all comparisons in bivariate analysis) dropped at multivariable analysis due to collinearity

The bold values show statistically significant p-values (p<0.05)

Factors independently associated with willingness to use HIVST were studying an arts-based college, being married or living with a partner, emergency contraceptive use, having at least one sexual partner, sexual experience, alcohol use, a history of unprotected sex with a partner of unknown HIV status, injection drug use, and sexually transmitted infections (STI) in the prior 12 months (p < 0.05 for all comparisons; Table 3). Arts-based college (aPR 0.92, 95% CI 0.88–0.97, p < 0.01), having one sexual partner (aPR 1.07, 95% CI 1.03–1.12, p < 0.01) or two or more partners (aPR 1.08, 95% CI 1.04–1.19, p < 0.01), alcohol use (aPR 1.04, 95% CI 1.00–1.09, p = 0.04), injection drug use (aPR 1.04, 95% CI 1.00–1.09, p = 0.04), having had an STI in 12 months (aPR 1.05, 95% CI 1.01–1.09, p = 0.02), and having tested for HIV in 12 months (aPR 1.12, 95% CI 1.02–1.22, p = 0.01) or more (aPR 1.13, 95% CI 1.03–1.24, p = 0.01) were significantly associated with willingness to use HIVST in multivariable analyses.

**Table 3:** Factors associated with willingness to use HIVST

Characteristic	Willing to use HIVST (N = 93), N (%)	Crude PR^a^ (95% CI)	p value	Adjusted PR^a^ (95% CI)	p value
College type					
Science	144 (96.0)	Reference		Reference	
Arts	300 (91.2)	0.95 (0.91–0.99)	**0.03**	0.92 (0.88–0.97)	** < 0.01**
Married or living with partner					
No	419 (92.3)	Reference			
Yes	25 (100.0)	1.08 (1.06–1.11)	** < 0.01**	-	-
Contraceptive use					
No	344 (91.7)	Reference			
Yes	100 (96.2)	1.05 (0.99–1.10)	0.06	-	-
Emergency pill in the past 6 months					
No	311 (91.2)	Reference			
Yes	133 (96.4)	1.06 (1.01–1.11)	**0.02**	-	-
Number of sexual partners					
None	204 (87.9)	Reference		Reference	
One	218 (96.9)	1.10 (1.04–1.16)	** < 0.01**	1.07 (1.03–1.12)	** < 0.01**
Two or more	22 (100.0)	1.14 (1.08–1.19)	** < 0.01**	1.08 (1.04–1.19)	** < 0.01**
Ever had sex					
No	203 (89.0)	Reference			
Yes	241 (96.0)	1.08 (1.02–1.14)	**0.01**	-	-
Alcohol use in past 3 months					
No	325 (91.0)	Reference		Reference	
Yes	119 (97.5)	1.07 (1.03–1.12)	** < 0.01**	1.04 (1.00–1.09)	**0.04**
Unprotected sex with > 1 partner of unknown HIV status					
No	417 (92.3)	Reference			
Yes	27 (100.0)	1.08 (1.06–1.11)	** < 0.01**	-	-
Injection drug use in past 6 months					
No	436 (92.6)	Reference		Reference	
Yes	8 (100.0)	1.08 (1.05–1.11)	** < 0.01**	1.04 (1.00–1.09)	**0.04**
STI^b^ in the past 12 months					
No	333 (91.0)	Reference		Reference	
Yes	111 (98.2)	1.08 (1.04–1.12)	** < 0.01**	1.05 (1.01–1.09)	**0.02**
Ever tested for HIV					
Never	83 (83.0)	Reference		Reference	
Yes, within 12 months	206 (95.8)	1.15 (1.05–1.27)	** < 0.01**	1.12 (1.02–1.22)	**0.01**
Yes, over 12 months	155 (94.5)	1.14 (1.03–1.25)	**0.01**	1.13 (1.03–1.24)	**0.01**
Ever used HIVST					
No	355 (92.0)	Reference			
Yes	89 (95.7)	1.04 (0.99–1.10)	0.14	–	–

^a^PR (Prevalence ratio);

^b^STI (Sexually Transmitted Infection);

Partner has other partners, partner is circumcised, partner provides financial and material support, partner’s HIV status, partner among populations at high risk, and age of partner (all p < 0.05 for all comparisons in bivariate analysis) dropped at multivariable analysis due to collinearity

The bold values show statistically significant p-values (p<0.05)

## Discussion

In this cross-sectional study of 483 female students at Makerere University, we found that majority of students (93%) were willing to self-test for HIV, despite the low uptake of HIVST (19%). Respondent’s age was associated with a history of HIVST use whereas college type, number of sexual partners, alcohol or drug use, STIs, and HIV testing experience predicted willingness to self-test for HIV.

We found that one-in-five female students had ever used HIVST, which is higher than the 9% reported among Nigerian university students [20]. Students from Uganda were assessed at least 2 years after those in Nigeria, and because HIVST is being scaled-up in sub-Saharan Africa, Ugandan students in this study may have had better access to HIVST. Oral self-test kits were freely available at the University Hospital during the study period and could be purchased over the counter in pharmacies near the university. However, self-test kit utilization by university students was modest (19%) and its scale-up may have been impeded by COVID-19-related lockdowns [21] that happened in Uganda in 2020 and 2021. Nonetheless, uptake was higher than anticipated given that Uganda implemented its HIVST policy in 2019 and recommended HIVST in the 2020 HIV clinical guidelines [11, 22].

Nearly all students were willing to use self-test kits when freely available. HIVST was highly acceptable among AGYW who could interpret their results accurately [14]. Correspondingly, studies conducted at tertiary learning institutions elsewhere in Africa found that 59–86% of female students were willing to self-test for HIV [13, 23–25]. Students at Makerere University received free access to self-test kits from the institution’s hospital, which could have influenced their desire to use HIVST. Leveraging on the high acceptability of self-test kits, delivery through trained peers at female student’s places of residence could increase HIVST uptake and demand for HIV testing services [13].

Only 80% of female students had ever been tested for HIV, significantly below UNAIDS 95–95-95 targets of 95% people knowing their HIV status [12]. It is likely that some students who had never been tested, such as the 47% who were sexually inexperienced did not see the necessity for HIV testing or had a low self-risk perception of HIV [13]. Notably, HIV testing among female university students was below the national average for AGYW (92% for older AGYW) in Uganda [8]. HIVST can bridge the gaps in HIV testing because it is highly acceptable to AGYW [14, 20]. Additionally, HIVST is crucial in reaching key populations and their partners, especially when access to health facilities is constrained, as was the case of COVID-19 lockdown [21].

Young women at university are more likely to use HIV self-test kits as they get older (aPR: 1.23 per year). A study among AGYW in Kenya found that older age (adjusted Risk Ratio: 1.09 per year) was associated with HIVST uptake [26]. It is possible that as female students spend more time at university, their awareness of HIVST and where to get self-test kits grows. Older students may have been more sexually active and had a higher HIV-risk perception, both of which may have influenced their decision to self-test for HIV [20]. Senior students can be trained and supported as peers to distribute HIVST kits to younger students during their freshman orientation and places of residence.

Arts students were less likely to be willing to self-test for HIV, whereas students who had sexual partners, used alcohol or injectable drugs, had an STI in the past year, or had ever tested for HIV were more willing to self-test for HIV. Arts students may be less willing to self-test for HIV than their science colleagues, due to their lower HIV knowledge and sexual risk perception [20]. Due to their sexual risk for HIV, students with sexual partners maybe not be using condoms consistently and may be influenced by and willing to self-test with their male partners [23, 24]. Multiple sexual relationships, alcohol and injection drug use and STIs are among many factors influencing young women’s susceptibility to HIV [3]. Young women who engage in these high risk behaviours have a higher risk perception and desire to test for HIV [27]. Finally, students who have ever tested for HIV were probably sexually active, required frequent HIV testing, and opted for self-test kits which were convenient, easy to administer, and ensured privacy [28]. Student’s risk perception and HIV testing experiences may affect their desire to use HIVST. Students who offered science courses, had sexual partners, engaged in high-risk behaviours, and had previously tested for HIV should be given priority when distributing HIV self-test kits since they were more eager to self-test for HIV. These students could be identified through risk screening tools available online and at institutional hospitals.

The high response rate, student diversity, and the use of multivariable regression to control for potential confounders are all strengths of this study. Our findings, however, have limitations. First, responses were self-reported, which made them vulnerable to recall and social desirability bias. Notably, social desirability is less common with self-administered online surveys than face-to-face interviews [29]. Nevertheless, we included detailed explanations of HIVST and assured participants of anonymity of their responses, which may have further lowered the likelihood of these biases. Second, selection, misclassification, and confounding bias may have distorted the accuracy of the findings. The non-probability sampling technique (quota sampling) and response exclusion may have resulted in selection bias. Although graphics (in the online questionnaire) were used to demonstrate oral self-testing, it is possible that some students misconstrued blood tests for oral self-tests. Residual confounding might have arisen from unstudied variables that could have been key confounders. For example, we did not test for HIV and other STIs due to the study design. Third, due to research design constraints, we were unable to offer or follow up with students who were willing to use HIVST to assess whether they eventually used the HIV self-test kits. Finally, this study was conducted during the COVID-19 lockdown period, which may have masked HIV risk and awareness, as well as HIVST utilization and translation.

## Conclusions

We found that most female students at Makerere University were eager to utilize HIV self-test kits, notwithstanding the low utility of HIVST. Eight out of ten female students had ever tested for HIV, below the UNAIDS 2025 target of 95%. Increasing access and use of HIV self-test kits could bridge the HIV testing gap among young women, particularly among female students who have never tested for HIV. Future studies should evaluate strategies to promote uptake and use of HIV self-tests by young women in sub-Saharan Africa, including students in tertiary institutions of learning.

## Data Availability

The de-identified dataset supporting the conclusions of this article is available at Mendeley Data repository: https://data.mendeley.com/ (https://doi.org/10.17632/dmbt2sjmvp.1).
